# Whole-genome resequencing reveals genetic diversity and adaptive evolution in Chinese honeybee (*Apis cerana cerana*) in Guizhou, China

**DOI:** 10.3389/fgene.2024.1352455

**Published:** 2024-05-17

**Authors:** Yinchen Wang, Bing Zeng, Mengqing Deng, Tian Zhao, Yan Liao, Rongqing Ren, Hua Wang, Yang Yuan

**Affiliations:** ^1^ Guizhou Institute of Animal Husbandry and Veterinary Science, Guiyang, China; ^2^ College of Animal Science and Technology, Southwest University, Chongqing, China

**Keywords:** *Apis cerana* cerana, whole-genome resequencing, genetic diversity, genetic structure, selective sweep

## Abstract

**Introduction:** Guizhou Province, characterized by complex and diverse geographic and climatic environments, has rich genetic resources for the Chinese honeybee (*Apis cerana cerana*) and is one of the main bee-producing areas in China. However, research on the genetic diversity of Chinese honeybee in the Guizhou region is very limited, despite implications for conservation of biodiversity.

**Methods:** In this study, we analyzed the genetic diversity, differentiation, and selection signals based on 116 Chinese honeybees from 12 regions in Guizhou Province using whole-genome sequencing.

**Results:** We identified 1,400,430 high-quality SNPs across all samples. A population structure analysis revealed two independent genetic subgroups of Chinese honeybees in Guizhou, a Yunnan-Guizhou Plateau population in western Guizhou and a hilly-mountainous population in eastern Guizhou. The average nucleotide diversity (*Pi*) ranged from 0.00138 to 0.00161 and average expected heterozygosity (*H_e_*) ranged from 0.2592 to 0.2604. The average genetic differentiation index (*F*_ST_) for Chinese honeybees in pairwise comparisons of 12 regions ranged from 0.0094 to 0.0293. There was clear genetic differentiation between the western plateau and the eastern hilly mountainous areas of Guizhou; however, *F*_ST_ values between the eastern and western populations ranged from 0.0170 to 0.0293, indicating a low degree of differentiation. A genome-wide scan revealed a number of genes under selection in the Yunnan-Guizhou Plateau environment. These genes were related to growth and development, reproduction, and cold resistance, and several candidate genes involved in environmental adaptation were identified, including *CTR*, *MAPK*, *MAST*, *HSF*, and *MKKK*.

**Discussion:** The results of the present study provide important theoretical bases for the conservation, evaluation, development, and utilization of genetic resources for Chinese honeybees in the Guizhou region and for further investigations of environmental adaptation and underlying mechanisms in the species.

## 1 Introduction

The Chinese honeybee (*Apis cerana cerana*) is one of the most important species in the genus *Apis* in China; it effectively utilizes sporadic nectar plants, has a long nectar-harvesting period, a strong collecting ability, and a high level of resilience and is a valuable indigenous genetic resource (Yang, 2001; Hou et al., 2016). Under the complex ecological conditions in China, Chinese honeybees are well adapted to flowering plants and climatic variation in different regions, thus playing an irreplaceable and extremely important ecological role relative to that of exotic bees (Zhou et al., 2021; Chen and Luo, 2023). Research has shown that even Chinese honeybee populations in limited geographic areas may have great genetic differences, resulting in high diversity and providing a material foundation for the development and utilization of Chinese honeybee genetic resources (Yin and Ji, 2013; Liu et al., 2016; Yu et al., 2019; Cao et al., 2021; Fang et al., 2022). Changes in the climate and environment, industrialization, pesticide implementation, and other factors have affected honeybee resources. Recent research has shown that the distribution of honeybees in China is now highly patchy. With the rise of the beekeeping industry, unrestricted introductions and hybridization have increased the difficulty of native Chinese honeybee conservation (Zhou et al., 2018; Chen and Luo, 2023). Therefore, the conservation of indigenous superior genetic resources is necessary to cope with climate change.

The restriction of gene flow leads to genetic differentiation. Studies of local adaptation are of fundamental importance for understanding how species evolve and genetically diversify and for predicting the effects of environmental change on species distributions (Montero-Mendieta et al., 2019). Guizhou Province in southwestern China is characterized by a variety of topographic landscapes, including plateaus, mountains, and hills, with diverse regional microclimate types (Chen et al., 2018; Fan et al., 2021; Qian, 2023). Traditional apiculture in China is rooted in the history of Guizhou Province, which is one of the main production areas of Chinese honeybees in the country (Wei et al., 2020). However, in recent years, the introduction of foreign bee colonies, artificial breeding, and mobile beekeeping have caused problems, such as the mixed status of native Chinese honeybee resources (Fan et al., 2021; Zhao et al., 2023). Therefore, it is particularly important to study the genetic diversity and environmental adaptation of native Chinese honeybees in Guizhou. The genetic diversity of Chinese honeybee populations in Guizhou has been evaluated based on morphological features, mitochondrial DNA, mitochondrial tRNA fragments, and microsatellite markers, revealing genetic differentiation between honeybee populations in the eastern and western parts of Guizhou (i.e., Yungui Plateau bees and Central China bees, respectively) (Xu, 1986; Yang, 2001; Zhao et al., 2022; Zhao et al., 2023). However, some scholars have obtained the opposite results, reporting a lack of genetic differentiation in the Guizhou Chinese honeybee population (Luo et al., 2015; Yu et al., 2017; 2021). The inconsistent results may be explained by the limited markers, sample size, and coverage or the use of low-resolution molecular markers and other methods. Therefore, the detailed population structure and genetic diversity of the Chinese honeybee in Guizhou has not been fully determined, although this is a key prerequisite for understanding adaptive evolution and developing effective conservation strategies for native genetic resources.

The publication of the Chinese honeybee genome atlas (Park et al., 2015; Diao et al., 2018; Wang et al., 2020) and whole-genome resequencing enable the identification of genetic variation in individuals and populations, improving the efficacy and resolution of traditional genetic methods and the identification of the molecular basis of region-specific adaptive and economic traits (Radwan and Babik, 2012). Genetic diversity studies covering the whole genome have higher reliability than that of studies based on traditional morphology and single molecular markers (Tang et al., 2022; Gao et al., 2023). However, the current understanding of the genome of the Chinese honeybee in Guizhou is very limited, and genomic differences between populations in different regions of Guizhou are not clear. In this study, we collected 116 samples of Chinese honeybees from 12 regions covering the eastern and western parts of Guizhou Province for whole-genome resequencing and investigated genetic diversity, population structure, and genetic differentiation. We performed the preliminary identification of key signaling pathways and candidate genes involved in environmental adaptation in Chinese honeybees from the western plateau of Guizhou in combination with an analysis of genomic signals of selection. The results of this study will be useful for the further establishment of strategies for conservation and environmental protection as well as the development and utilization of the genetic resources of the native Chinese honeybees in Guizhou Province.

## 2 Materials and methods

### 2.1 Sample acquisition

**FIGURE 1:**
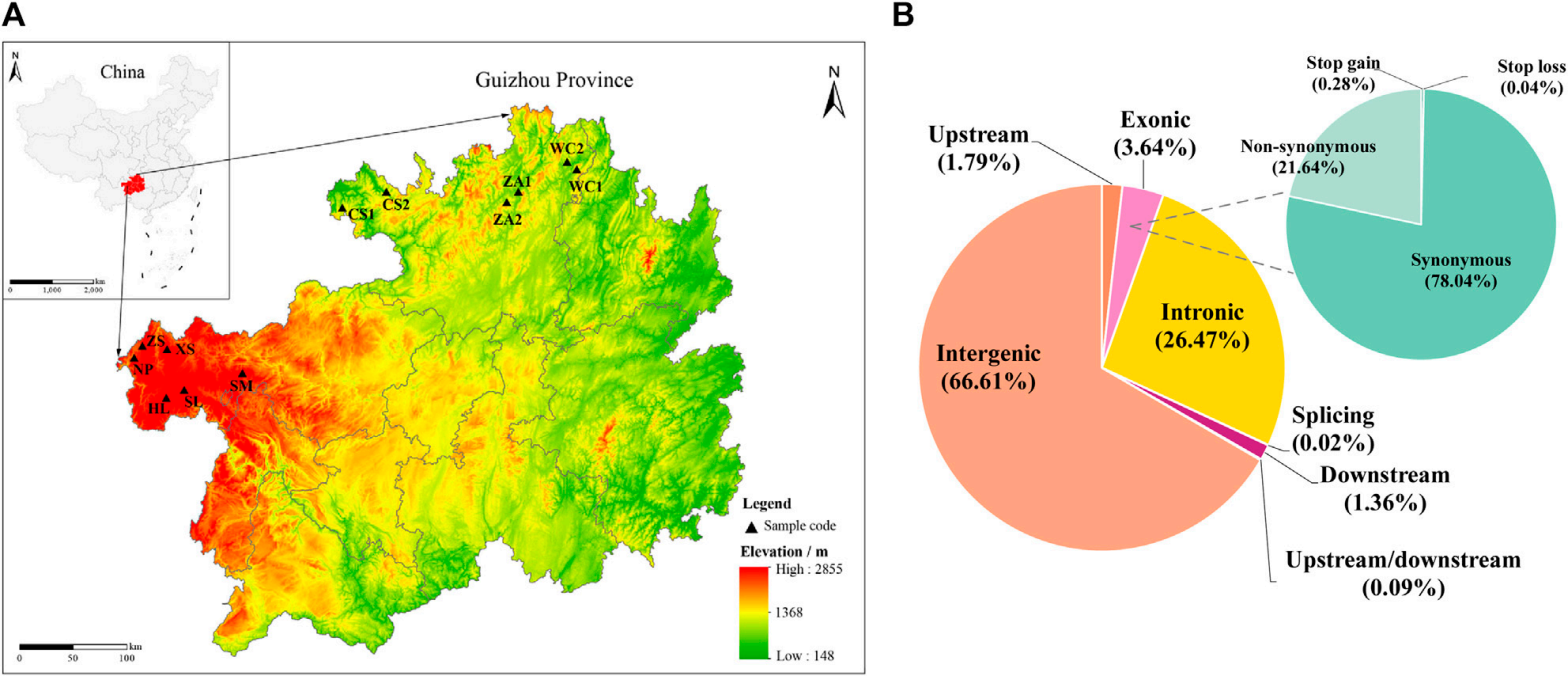
Distribution of samples collected from Chinese honey bees in Guizhou and distribution of SNPs in the Chinese honey bee genome. **(A)** Sampling locations and geographical distribution of Chinese honey bee colonies in 12 regions. **(B)** Distribution of SNPs detected in the genomes of Chinese honey bee colonies from 12 regions. The position of the SNPs in gene structures (lower left) and annotations of the SNPs in the exons (upper right). NP, Niupeng; ZS, Zhongshui; HS, Heishi; XS, Xueshan; SM, Shimen; SL, Shilong; CS1, Chishui 1; CS2, Chishui 2; ZA1, Zheng’an 1; ZA2, Zheng’an 2; WC1, Wuchuan 1; WC2, Wuchuan 2.

Samples were collected from six regions in the highland mountainous areas of western Guizhou, including Niupeng (NP), Zhongshui (ZS), Heishi (HS), Xueshan (XS), Shimen (SM), and Shilong (SL), as well as from six regions in the lower hilly mountainous areas of eastern Guizhou, including Chishui (CS1, CS2), Zheng’an (ZA1, ZA2), and Wucheon (WC1, WC2) (Figure 1A; Supplementary Table S1). In addition to the six samples collected from the Xueshan honeybee colony, 10 samples were collected from each area, each sample from a separate colony, totaling 116 Chinese honeybees (*Apis cerana*). Samples were sampled from healthy colonies of bees kept in live frames with normal colony dynamics and without artificial queens. These colonies were from natural sub-colonies of wild bees and were in a wild and semi-wild state (domestic and wild bees are frequently exchanged). Samples were taken from randomly selected worker bees within these colonies. The samples were treated with anhydrous ethanol and stored in an ultra-low temperature refrigerator at −80°C for further DNA extraction and sequencing.

### 2.2 DNA extraction and resequencing library construction

Total genomic DNA was extracted from the thoracic tissues of individual honeybees using the universal Genomic DNA Kit (Cat. No. CW0553, ComWin Biotech, Beijing, China) according to the manufacturer’s protocols. The genomic DNA was solubilized in TE buffer, and the samples were examined for degradation and impurities by agarose gel electrophoresis (agarose gel, 1%). The purity and concentration of genomic DNA were determined using a NanoDrop One Ultra-microspectrophotometer (Thermo Scientific, Waltham, MA, United States). Approximately 1 μg of genomic DNA template was obtained, and a resequencing library was prepared according to the procedure described in the TruSeq DNA Sample Preparation Guide (Illumina, 15026486 Rev. C). The DNA was randomly fragmented by ultrasonic crushing, and 350 bp fragments were selected for end-repair and 3′-end A-addition, followed by the addition of adapter sequences and enrichment for the target fragments by PCR. The qualified libraries were subjected to paired-end sequencing (PE150) on the HiSeq X Ten sequencing platform by Beijing Ovison Genetics Technology Co. Clean reads were obtained by filtering the raw reads; sequences with adaptors, sequences with an N content of >10%, and low-quality reads (in which bases with Q < 20 accounted for more than 50% of the whole read) were removed.

### 2.3 Sequence alignment and SNP calling

The published genome sequence of the Chinese honeybee (*Apis cerana*) [GenBank no. GCA_002290385.1] (Diao et al., 2018) was used as a reference sequence, and all nomenclature and annotation results were used as a reference. High-quality data obtained from resequencing were compared with the reference genome sequence using BWA (Li and Durbin, 2009) with the parameters mem “-t 4 -k 32 –M.” Redundant reads were filtered using SAMtools (Li et al., 2009) (parameters: rmdup). By comparing the positions of clean reads on the reference genome, the sequencing depth and genome coverage for each sample were determined. GATK (v4.4.2) (Quinlan et al., 2010) was used to detect SNPs in the samples and to filter low-quality SNPs. To ensure the accuracy of the test results, the filtering conditions were set as follows: the coverage depth of SNPs was more than 3, the proportion of deletions was less than 10%, and the minimum allele frequency was more than 5%. Other variant filtering parameters were set to officially specified default values of GATK. The SNPs were annotated using ANNOVAR (Wang et al., 2010).

### 2.4 Genetic structure analysis

TreeBeST-1.9.2 (Yang, et al., 2011) was used to obtain pairwise distance matrices for all individuals as a basis for constructing a phylogenetic tree of 116 samples by the neighbor-joining (NJ) method. Bootstrap values were calculated based on 1,000 replicates. Admixture software (Alexander, et al., 2009) was used to analyze the population genetic structure of Chinese honeybees in 12 regions. The number of subpopulations (K value) was predefined as 2–10 for clustering, and the clustering results were cross-validated to determine the optimal number of clusters based on the lowest cross-validation error. Based on SNP data, a principal component analysis (PCA) was performed for 116 individuals using GCTA (Yang et al., 2011).

### 2.5 Analysis of genetic diversity

Observed heterozygosity (*H*_*o*_) and expected heterozygosity (*H*_*e*_) were calculated using PLINK (Purcell et al., 2007). Genetic diversity indicators, such as nucleotide diversity (*Pi*) and polymorphism information content (*PIC*), were calculated and analyzed using Perl scripts (Wang, 2022). Genetic differentiation (*F*_*ST*_) was calculated using Genepop (Diniz-Filho et al., 2013). To assess the Linkage disequilibrium (LD) pattern, we calculated the squared allele frequency correlation (*r* ^2^) using Haploview 4.2 (Barrett et al., 2005). Additionally, LD decay graphs were generated using an R script (Chan, 2018).

### 2.6 Historical effective population sizes analysis

The PMSC method was employed, with the mutation rate set at 5.3 × 10^−9^ and the generation time estimated to be 1 year (Chen et al., 2016; Li and Durbin, 2011).

### 2.7 Selective sweep analysis

Currently, the central China-type Chinese honeybee is the most widely distributed ecotype among nine ecotypes in China and thus has been studied more extensively compared with the YunGui Plateau-type honeybee. We performed a genome-wide selection scan of the eastern Guizhou honeybee population using the western honeybee population as a reference. The log_2_ ratio of polymorphism (*Pi*_Eastern_/*Pi*_Western_) and the coefficient of population differentiation (*F*_ST_) for the two populations were calculated using the sliding-window method (40 kb sliding window, 20 kb step), with the top 5% of windows as the screening criterion (Chen et al., 2016; Su, 2023). Genes within the selected regions were extracted and subjected to Gene Ontology (GO) and Kyoto Encyclopedia of Genes and Genomes (KEGG) enrichment analyses using eggNOG-mapper (Cantalapiedra et al., 2021).

## 3 Results

### 3.1 Whole-genome resequencing and SNP calling

In this study, 116 Chinese honeybee population samples from 12 geographic sample sites in Guizhou were collected for whole-genome resequencing. After quality control, 270.11 Gb of clean reads were obtained. The average sequencing quality value of the samples from the 12 geographic populations was Q30 ≥ 91.12%, and the GC content ranged from 32.54% to 35.02%. The whole-genome resequencing data for the 116 honeybee colony samples were aligned with the reference genome sequences. The average sequencing depth was ×8.96, the average genome coverage was 97.68%, and the average comparison rate was 95.94% (Supplementary Table S2).

After rigorous quality screening, we obtained 1,400,430 high-quality SNPs in Chinese honeybee populations from 12 geographic regions. In total, 934,150 (66.70%) SNPs were located in intergenic regions, while 421,724 SNPs were located in genes, including 50,981 (3.64%) SNPs in exons and 370,743 SNPs in introns, accounting for 26.47% of the overall number (Figure 1B; Supplementary Table S3). In conclusion, most of the variants were located in non-coding sequences, including intergenic and intronic regions, suggesting that non-coding sequences with the potential to alter protein function by regulating gene expression contributed to adaptive evolution in honeybees.

### 3.2 Genetic structure

**FIGURE 2:**
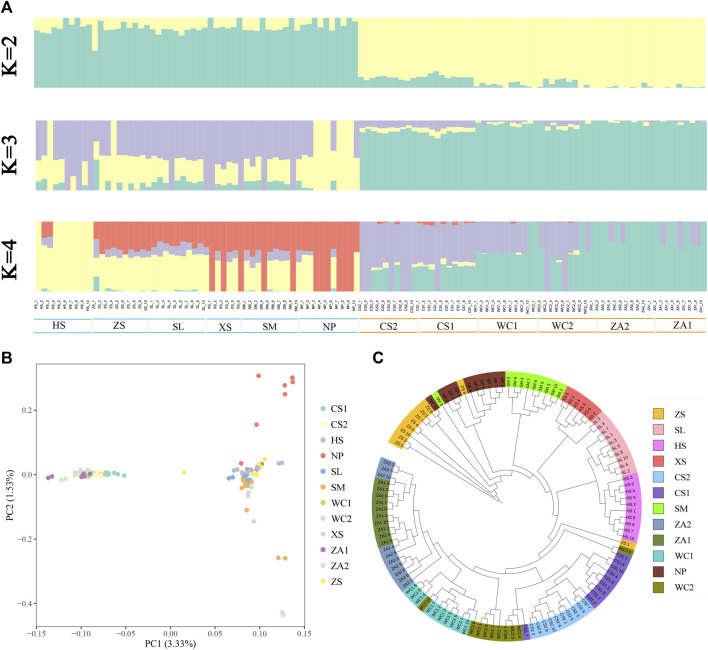
Analysis of the genetic structure of Chinese honey bee populations. **(A)** Clustering of Chinese honey bee populations using ADMIXTURE with K from 2 to 4. The colors represent proportions of samples in each of the K inferred clusters. **(B)** PCA with first (PC1) and second (PC2) principal components. **(C)** Phylogenetic tree of 116 Chinese honey bees in Guizhou. NP, Niupeng; ZS, Zhongshui; HS, Heishi; XS, Xueshan; SM, Shimen; SL, Shilong; CS1, Chishui 1; CS2, Chishui 2; ZA1, Zheng’an 1; ZA2, Zheng’an 2; WC1, Wuchuan 1; WC2, Wuchuan 2.

Admixture results showed that when assuming that two subpopulations were present in the samples (K = 2), honeybee populations from the western highlands (NP, HS, SM, SL, XS, and ZS) were clearly differentiated from populations from eastern low hilly mountainous populations (WC1, WC2, ZA1, ZA2, CS1, and CS2) (Figure 2A). Except for CS1, CS2, and a few populations from the western highland region with relatively pure ancestries, the honeybee populations from the remaining regions showed mixed ancestries to varying degrees, implying that there is a certain degree of genetic similarity between the eastern and western bee populations. The populations from the NP and HS regions were separated when setting K = 3 and K = 4. The cross-validation error rate was minimized (Supplementary Table S4) when the K value was 2, indicating that this was the optimal K value. A PCA and NJ tree analyses further supported the two genetic groups (Figures 2B, C; Supplementary Figures S1, S2), reflecting the geographical distribution. These findings indicate that Chinese honeybee populations in the western plateau and the eastern hilly mountainous areas of Guizhou belong to two independent genetic groups, respectively.

### 3.3 Genetic diversity

Nucleotide diversity, an indicator of intra- or inter-population diversity, ranged from 0.00138 to 0.00161 for the different populations, with the highest average *Pi* values for the HS and CS2 populations (0.00161) and the lowest for the ZA2 population (0.00138) (Table 1). The overall *PIC* of the 12 regional populations was 0.2855, on average, indicating moderate polymorphism (0.25 < *PIC* < 0.5) (Botstein et al., 1980). The expected heterozygosity for honeybee populations in each region ranged from 0.2592 to 0.2604, and the observed heterozygosity ranged from 0.2456 to 0.2506 (Table 1). The expected heterozygosity (mean *H*_*e*_ = 0.2596) of populations in different regions was higher than the observed heterozygosity (mean *H*_*o*_ = 0.2487), and the overall mean heterozygosity of the populations was lower than 0.5 (Leberg, 2002), suggesting that heterozygosity and genetic diversity were low in the Chinese honeybee in different regions of Guizhou. The results of the LD analysis showed that the XS population had the slowest rate of decay, followed by the ZA2 population, with the other populations decaying at similar rates (Supplementary Figure S3).

**TABLE 1:** Genetic diversity of Chinese honey bees in 12 regions of Guizhou.

Sample code	He	Ho	PIC	Pi
ZS	0.2598	0.2550	0.2826	0.00158
SL	0.2600	0.2489	0.2817	0.00160
HS	0.2601	0.2504	0.2834	0.00161
XS	0.2593	0.2493	0.3140	0.00160
CS2	0.2597	0.2506	0.2818	0.00161
CS1	0.2593	0.2472	0.2813	0.00158
SM	0.2595	0.2499	0.2830	0.00160
ZA2	0.2604	0.2443	0.2830	0.00138
ZA1	0.2593	0.2471	0.2862	0.00156
WC1	0.2592	0.2456	0.2827	0.00153
NP	0.2597	0.2495	0.2838	0.00157
WC2	0.2593	0.2466	0.2862	0.00156

Note: He: expected heterozygous number; Ho: observed heterozygous number; Pi: nucleic acid diversity; PIC: polymorphism information content. The sampling place codes are NP, Niupeng; ZS, Zhongshui; HS, Heishi; XS, Xueshan; SM, Shimen; and SL, Shilong; CS1, Chishui 1; CS2, Chishui 2; ZA1, Zheng’an 1; ZA2, Zheng’an 2; WC1, Wuchuan 1; WC2, Wuchuan 2.

### 3.4 Genetic differentiation

**FIGURE 3:**
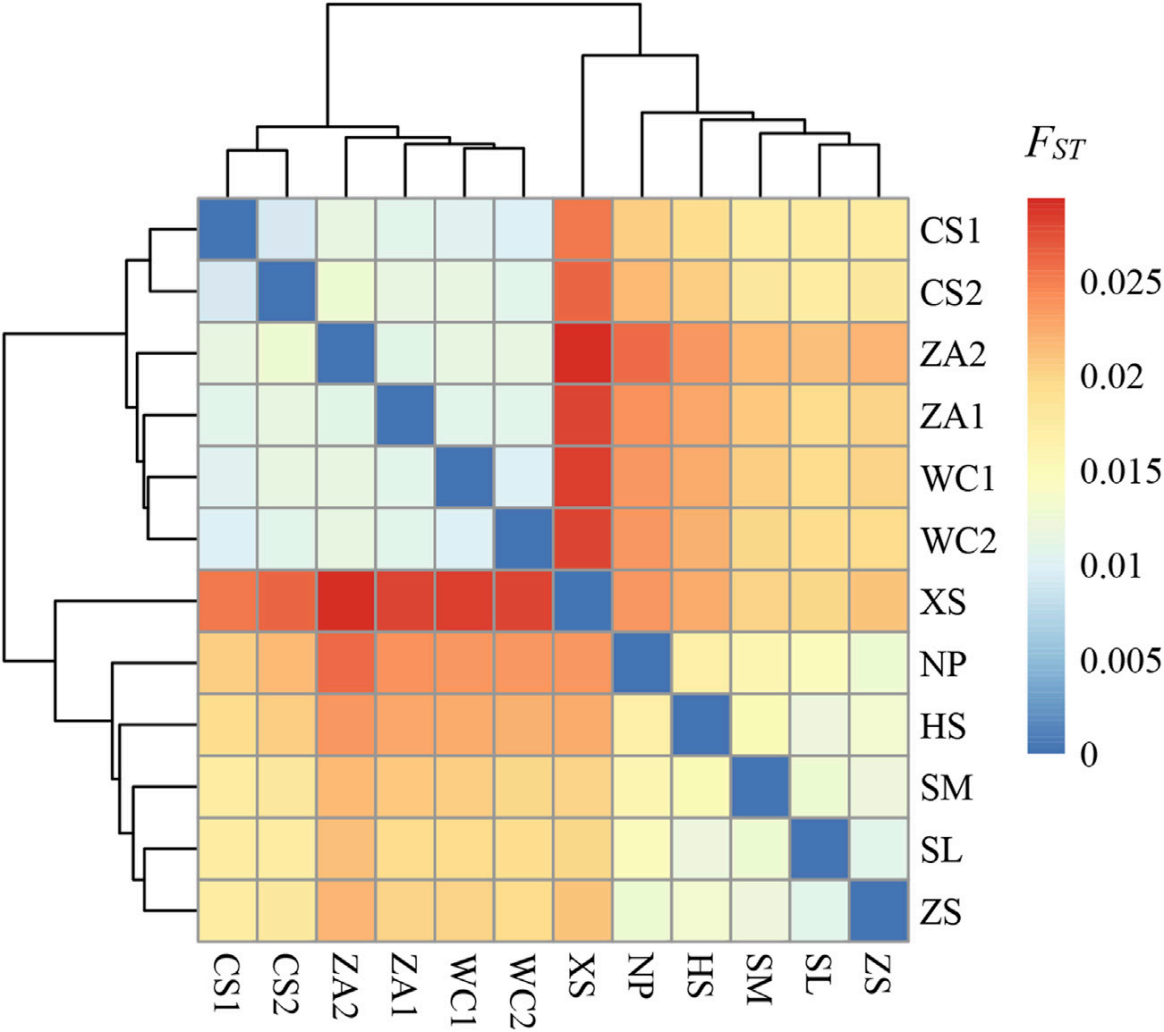
Clustered heat map of genetic differentiation indices of honey bee populations in 12 regions. The color of the squares in the figure reflects the magnitude of the *F*_*ST*_ values, with darker colors indicating a higher degree of genetic differentiation between the two populations of bees intersecting horizontally and vertically. NP, Niupeng; ZS, Zhongshui; HS, Heishi; XS, Xueshan; SM, Shimen; SL, Shilong; CS1, Chishui 1; CS2, Chishui 2; ZA1, Zheng’an 1; ZA2, Zheng’an 2; WC1, Wuchuan 1; WC2, Wuchuan 2.

With respect to genetic differentiation between populations of Chinese honeybees, paired *F*_*ST*_ values of the 12 geographic groups ranged from 0.0094 to 0.0293, with an average of 0.0182. In particular, the XS and ZA2 populations showed the highest differentiation (*F*_ST_ = 0.0293), while to the two eastern populations CS1 and CS2 showed the lowest differentiation (*F*_*ST*_ = 0.0094) (Table 2). By region, paired *F*_ST_ values between the western groups and each of the eastern groups was 0.0170–0.0293, with a mean of 0.0218, indicating weak genetic differentiation between the eastern and western groups (0 < *F*_ST_ < 0.05). A clustering heat map based on *F*_ST_ values demonstrated genetic differentiation between the eastern and western populations (Figure 3), and the clustering results were consistent with the results of the genetic structure analyses, further indicating that there was obvious genetic differentiation between the eastern and western populations of Chinese honeybees in Guizhou.

**TABLE 2:** Pairwise genetic differentiation coefficients (*F*_*ST*_) among 10 populations of the *Apis cerana*.

	CS1	CS2	HS	NP	SL	SM	WC1	WC2	XS	ZA1	ZA2	ZS
CS1	—											
CS2	**0.0094**	—										
HS	0.0193	0.0204	—									
NP	0.0204	0.0217	0.0168	—								
SL	0.0170	0.0172	0.0119	0.0145	—							
SM	0.0173	0.0182	0.0151	0.0156	0.0126	—						
WC1	0.0104	0.0115	0.0225	0.0237	0.0195	0.0203	—					
WC2	0.0099	0.0106	0.0221	0.0235	0.0192	0.0197	0.0100	—				
XS	0.0255	0.0263	0.0223	0.0237	0.0198	0.0200	0.0282	0.0280	—			
ZA1	0.0106	0.0117	0.0227	0.0239	0.0196	0.0207	0.0108	0.0107	0.0279	—		
ZA2	0.0117	0.0127	0.0235	0.0258	0.0212	0.0217	0.0115	0.0115	**0.0293**	0.0111	—	
ZS	0.0173	0.0179	0.0130	0.0129	0.0106	0.0122	0.0201	0.0196	0.0211	0.0201	0.0217	—

Note: The sampling place codes are NP, Niupeng; ZS, Zhongshui; HS, Heishi; XS, Xueshan; SM, Shimen; and SL, Shilong; CS1, Chishui 1; CS2, Chishui 2; ZA1, Zheng’an 1; ZA2, Zheng’an 2; WC1, Wuchuan 1; WC2, Wuchuan 2. Bold font indicates the two largest (0.0293) and smallest (0.0094) values of the pairwise genetic differentiation coefficients for the 10 colonies, respectively.

### 3.5 Historical effective population sizes

The PSMC model is a method for estimating changes in effective population size with high reliability (Li et al., 2012). In order to understand the important evolutionary events that occurred in the past adaptation process of different populations, we estimated their historical effective population sizes using the PSMC method (Supplementary Figure S4). According to the estimation results, the effective population sizes of Chinese honey bee populations in 12 geographic regions were almost the same around 800,000–500,000 years ago, while around 400,000 years ago, the effective population sizes of these populations gradually increased and began to show different population size variations among groups. The effective population size peaked around 130,000 to 110,000 years ago (a relatively warm geological period). After that, the population size continued to decline on a large scale, reaching its lowest point during the Last Glacial Maximum (about 15,000 years ago), and then the population recovered again. Overall, the effective population size histories of Chinese honey bee populations in the 12 geographic regions of Guizhou showed similar patterns.

### 3.6 Selective sweep analysis

**FIGURE 4:**
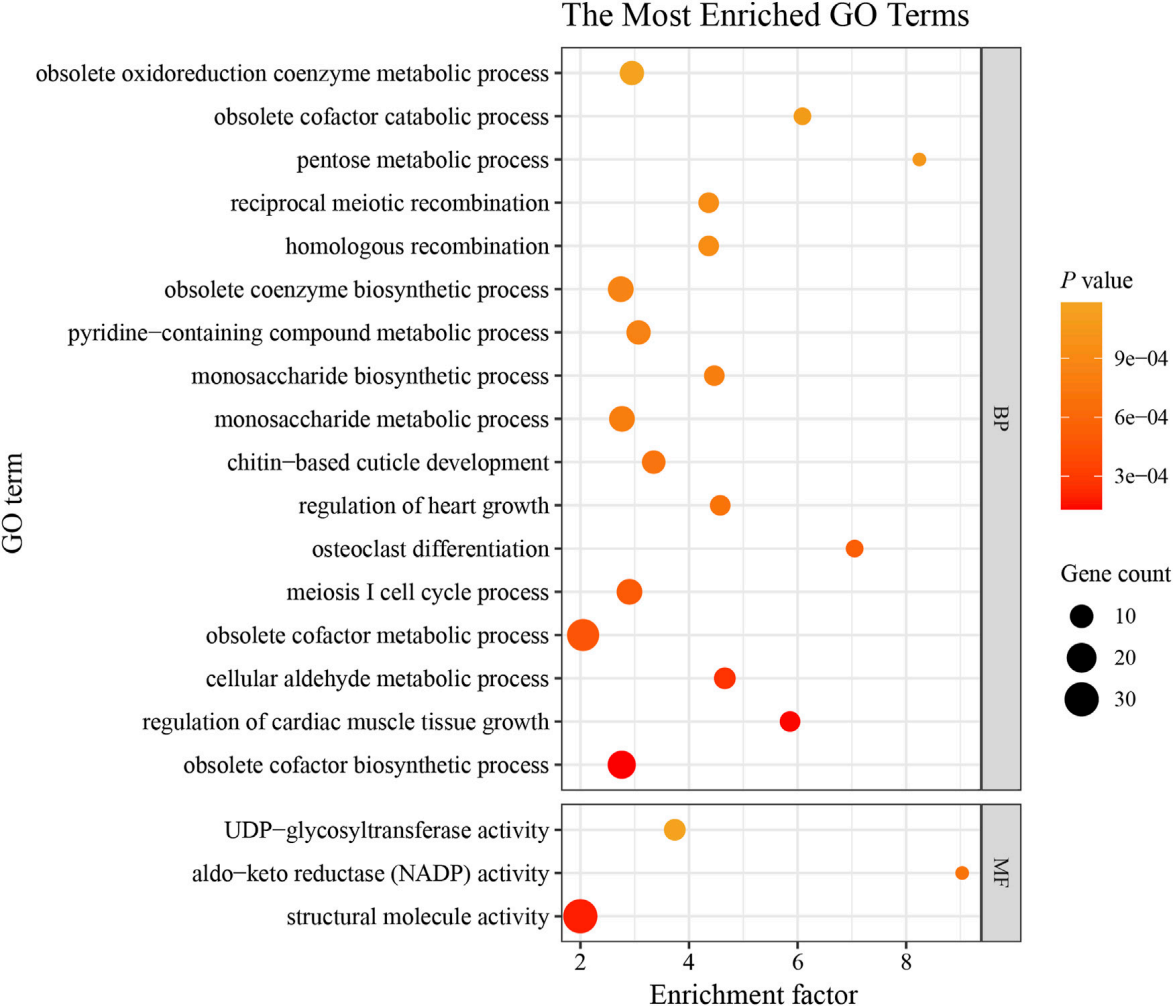
Top 20 GO terms with significant enrichment. BP, biological process; MF, molecular function. The sizes of the bubbles indicate the number of genes enriched in the GO term. The color of the bubble represents the *p*-value. The enrichment factor is the ratio of selected gene numbers annotated in this GO term to all gene numbers annotated in this GO term. The greater the rich factor is, the greater the degree of GO term enrichment.

We screened the genomic regions potentially subject to selection in Chinese honeybees in western Guizhou using both *Pi* ratios and *F*_ST_ values, using eastern honeybees as the reference population (Supplementary Figure S5). A total of 184 potentially selected regions and 369 potentially selected genes were identified (Supplementary Table S5). To further analyze the functions of these candidate genes, they were subjected to functional enrichment analyses using the GO with KEGG databases. A total of 289 GO entries and 11 KEGG metabolic pathways were significantly enriched (*p* < 0.05) (Supplementary Tables S6, S7). The GO enrichment analysis showed that the selected genes functioned in “osteoclast differentiation,” “meiosis I cell cycle process,” “regulation of cardiac muscle tissue growth,” and “positive regulation of intracellular signal transduction” (Figure 4; Supplementary Table S6), suggesting that these genes under selection are involved in reproduction, growth and development, and environmental adaptation in Chinese honeybee colonies in the western plateau region. In a KEGG pathway enrichment analysis, the genes were involved in “Pentose and glucuronate interconversions,” “Glycerolipid metabolism,” and “Biosynthesis of amino acids,” indicating enrichment for the metabolic pathways of sugars, lipids, and amino acids (Supplementary Figure S6; Supplementary Table S8). The evolution of functional genes related to the metabolism and synthesis of sugars, lipids, and amino acids plays a key role in improving energy metabolism in the Chinese honeybee. In addition, “Terpene skeleton biosynthesis” may also be closely related to olfactory learning and memory in honeybees (Huang et al., 2023). Functional enrichment analysis based on selected genes, we identified calcitonin receptor (*CTR*, *APICC_06505*, *APICC_06502*), Mitogen-activated protein kinase (*MAPK*, *APICC_00669*), Microtubule-associated serine/threonine-protein kinase (*MAST*, *APICC_03515*), Mitogen-activated protein kinase kinase kinase (*MKKK*, *APICC_02754*), and Heat shock factor (*HSF*, *APICC_09465*) (Supplementary Tables S5, S8) as key candidate genes for adaptation to the western plateau region. These genes may be related to the biological characteristics of the Yungui Plateau-type Chinese honeybees, such as cold resistance, strong egg-laying ability, and high nectar production, which are related to reproduction, growth, development, and other physiological processes.

## 4 Discussion

### 4.1 Genetic structure and genetic diversity

Xu (1986) previously detected morphogenetic differentiation between eastern and western populations of the Chinese honeybee in Guizhou Province; the western populations were distributed in the northwestern region centered on Weining (Caohai) in Guizhou Province (the Yunnan-Guizhou Plateau ecotype) and the eastern populations were distributed in the region east of the “Zijin-Zhenning-Zhuheng” boundary (central China ecotype). The Chinese honeybees between the two regions represent a transitional form between the two ecotypes. Studies based on morphology combined with mitochondrial DNA and microsatellite markers have yielded similar results (Zhao et al., 2022; Zhou et al., 2023). In this study, we focused on the core distributions of these two established ecotypes of honeybees and performed a fine-scale genetic structure analysis at the genome-wide level (including 116 individuals of Chinese honeybee species from 12 regions). We corroborated previous findings at the genome-wide level based on population structure, phylogenetic analyses, and PCA (Figure 1) (Zhao et al., 2022; Zhou et al., 2023). Although the sampling areas in this study were relatively limited, they generally reflect the genetic backgrounds of the two typical extant ecotypes of Chinese honeybees in Guizhou Province. The results of this study also provide a database for expanding the scope of genetic analyses and for the conservation of genetic resources.

Measuring population genetic diversity provides insight into the current status and future trends of genetic resources, which is critical for genetic resource conservation and exploitation (Xu, 2021). Honeybees are social insects and respond appropriately to external environmental changes. High levels of population genetic diversity enable adaptation to a broad range of conditions, including extreme environments. High diversity provides a more stable internal environment within a colony, high productivity, and high disease resistance under environmental stress (Tarpy, 2003; Jones et al., 2004; Mattila and Seeley, 2007). In this study, estimates of overall mean nucleotide diversity in Chinese honeybee populations in the 12 regions of Guizhou (Table 1) were similar to those of populations in the Tibetan Plateau region (*Pi* = 0.00102–0.00155) (Tang et al., 2022) and lower than those in Yunnan (*Pi* = 0.0028–0.0039) (Montero-Mendieta et al., 2019). Analyses of *PIC*, *H*_*o*_ *,* and *H*_*e*_ (Table 1) all indicated that the genetic diversity in the Chinese honeybee population in Guizhou was low. The results of the LD analysis of the groups showed that the decay curves were similar, except for the ZA2 and XS groups, which showed a more pronounced population differentiation, indicating that the different groups were less differentiated. With global warming and the frequent occurrence of extreme weather, rich genetic resources are an important basis for coping with climate change. Therefore, it is imperative to focus on conserving the genetic structure and improving the genetic diversity of local Chinese honeybee genetic resources (Zhao et al., 2022).

### 4.2 Population genetic differentiation

*F*_ST_ is an important indicator of the degree of genetic differentiation between populations. According to Wright (1978), values of *F*_ST_ below 0.05 indicate low genetic differentiation and values between 0.05 and 0.15 show moderate differentiation. Our data showed that the average paired *F*_*ST*_ value of the 12 populations was 0.0182 (Table 2). The level of genetic differentiation was lower than estimates in the Tibetan Plateau region (mean *F*_ST_ = 0.0758) (Tang et al., 2022) and North China (mean *F*_ST_ = 0.132) (Yancan et al., 2019) and was similar to estimates in Central China (mean *F*_ST_ = 0.02470) (Fang et al., 2022) and Yunnan (mean *F*_ST_ = 0.027) (Montero-Mendieta et al., 2019). The mean *F*_*ST*_ value between the eastern and western populations was 0.0218, which was higher than the overall mean (0.0182), indicating that there was a certain degree of genetic differentiation between the two geographic regions (Table 2; Figure 3). The low level of genetic differentiation may be related to gene flow between the eastern and western populations (Xu, 1986). This also suggests that although the overall genomes of the eastern and western Chinese honeybee populations are similar, they are clearly shaped, in part, by selection. Similarly, a molecular marker-based study revealed moderate genetic differentiation between eastern and western Chinese honeybee populations (*F*_ST_ = 0.04 for the tRNA^leu^–COIl fragment and *F*_ST_ = 0.07 for the COI fragment) (Zhou et al., 2023). The relatively low *F*_ST_ values in this study may be attributed to the focus on the average nucleotide diversity across the whole genome, without emphasis on regions under strong selection. In conclusion, we further demonstrated that the eastern and western Chinese populations in Guizhou region are genetically differentiated at the genome level to some extent and belong to two relatively independent ecotypes.

Populations in different environments face different selection pressures. Elevation is an important environmental parameter; populations at higher elevations must adapt to various factors, such as higher radiation, lower temperatures, precipitation, and differences in plant phenological periods than those at lower elevations (Montero-Mendieta et al., 2019; Fang et al., 2022; Chen and Luo, 2023). The Guizhou region shows a gradual elevation from east to west and does not have features associated with geographic isolation, such as obvious islands and steep mountain ranges (Zhou et al., 2023). Honeybees have rapid migratory ability and, in the absence of barriers, geographic distance has little effect on population differentiation (Ji et al., 2009; Yin et al., 2011; Yancan et al., 2019). Therefore, we hypothesize that local ecological differences are an important cause of differentiation. It has been suggested that the nuptial flights of the Chinese honeybee are restricted to a certain altitude range, avoiding the decline in the fitness of offspring due to genes from different altitudinal environments (Luo, 2021; Zhu et al., 2022). The average altitude of the six sampling areas in eastern Guizhou was about 800 m. The topography of the area was mostly mountainous and hilly with karstic terrain. The average annual temperature was mostly at 20°C, and the average annual rainfall was about 1,100 mm, with a humid climate in the middle-subtropical zone, and the pollen source vegetation was widely distributed and diverse (Zhao et al., 2022). In contrast, the average elevation of the six sampling areas in the west was about 2,200 m above sea level, especially in the HS and XS areas, which are located in the Wumeng Mountains in the center of the Yunnan-Guizhou Plateau (one of the four major plateaus in China). The average annual temperature is only about 13°C, and the average annual rainfall is about 940 mm, belonging to the warm-temperate temperate climate zone of the plateau mountains. The region has a high degree of rocky desertification, a relatively limited and sporadic distribution of vegetation, and a later flowering period compared to that in the eastern region. The overwintering period of bee colonies in the western high-altitude areas is relatively longer during the cold period in the winter and spring seasons (Xu, 1987; Wei et al., 2016; Su, 2021). Therefore, there are obvious ecological differences between eastern and western Guizhou, and Chinese honeybee populations in each of these regions have been undergoing adaptive evolution independently for a long period of time, leading to population differentiation. Estimates of effective population sizes suggest that this difference may have first originated between about 130,000 and 110,000 years ago, at the onset of the last glacial period of the fourth season (Clark, et al., 2009; Batchelor, et al., 2019). When the ice age comes, populations living at higher elevations may be subject to stronger selection. Similarly, for example, populations of Chinese honeybees distributed in low- and high-altitude plateaus in western Sichuan (Zhu et al., 2022.), Gansu (Xi et al., 2012), Yunnan (Montero-Mendieta et al., 2019), and the Qinghai-Tibetan Plateau (Tang et al., 2022) are also differentiated. Overall, we believe that local ecological differences, including differences in nectar plant resources, climate, altitude, and geographic location, impede gene flow in Chinese honeybees (Hodkinson, 2005; Hu et al., 2022; Zhu et al., 2022), and this requires further investigations in the future.

### 4.3 Adaptive evolution

Bees inhabiting different regions often exhibit different phenotypes and behavioral adaptations. Previous studies have found that western Yungui Plateau-type Chinese honeybees have a larger size, darker body color, and longer villi than those of eastern Central China-type populations (Lin et al., 2018). Chinese honeybees at elevations above 2000 m in Yunnan Province also show distinct features (Zhang et al., 2014). Chinese honeybee populations at higher altitudes in Guizhou possess an outstanding overwintering ability, higher reproduction ability, and greater nectar collection and honey storage abilities than those of populations at lower altitudes (Xu, 1987; Luo et al., 2015). Seasonal dynamics, nectar collection behaviors, and egg-laying ability, which are population-level biological characteristics adapted to the climate of origin, persisted in these different ecotypes of Chinese honeybees even when they were reared *ex situ* (Xu et al., 2018a). These results also imply that biological traits shaped by long-term adaptation to local climatic and resource conditions are largely heritable in Chinese honeybee ecotypes.

The Chinese honeybees in this study were all obtained from indigenous colonies under traditional breeding conditions under random mating in natural environments, with little influence of domestication. Therefore, the sampling strategy is reliable for understanding the evolution of Chinese honeybee populations and environmental adaptation under the special geographic conditions of Guizhou. Long-term natural selection often leads to changes in allele frequencies at selected loci and linked loci, and the identification of selected genomic features can help to reveal the genetic mechanisms of important traits for environmental adaptation in populations (Zhang et al., 2023). We used a combination of two methods for the detection of signals of selection (the intersection of the top 5% of *F*_*ST*_ values and *Pi* ratios) to screen the eastern and western (as a reference population) Chinese honeybee populations, and we annotated the selected genes and performed enrichment analyses to evaluate gene functions, which can help us to better understand the evolutionary mechanisms of Chinese honeybee adaptation in the Yungui Plateau.

#### 4.3.1 Genes related to growth and development

We detected enrichment for many functions, including “osteoclast differentiation,” “regulation of cardiac muscle tissue growth,” and “structural molecule activity” (Figure 4; Supplementary Tables S6, S8), suggesting that the candidate genes may contribute to the relatively large body size of populations in the western plateau. Plants are influenced by the climatic conditions of the region, generally showing shorter flowering periods and lower abundances in high alpine regions than at low altitudes (Cleland et al., 2007). With the increase of altitude, the depth of nectary gradually deepened (Montero-Mendieta et al., 2019). Therefore, Chinese honeybees at high altitudes often need to fly long distances to gather food. Many studies have shown that changes in body size-related traits, such as the wing area, femur length, tibia length, metatarsus length and width, and tongue length, of honeybees are significantly and positively correlated with increasing altitude (Tan et al., 2005; Zhang et al., 2014; Zhu et al., 2022). Increases in the sizes of the wing, hindfoot, and snout facilitate longer flights and more efficient pollen and nectar collection and transport, and a larger body size facilitates adaptation to cold (Scharf et al., 2014). Interestingly, we identified two genes encoding calcitonin receptor proteins (*APICC_06505* and *APICC_06502*), related to multiple growth and development-related GO entries. Calcitonin is a peptide hormone that promotes the proliferation and differentiation of osteoblasts. The calcitonin receptor is a G-protein-coupled receptor located on osteoblast membranes that promotes bone development by specifically binding to calcitonin, regulating calcium and phosphorus metabolism, and maintaining bone metabolic homeostasis (Pondel, 2000). Calcitonin receptors also play an important role in thermoregulation in *Drosophila* (Goda et al., 2018). The growth of wings, femoral, tibial, and basitarsal segments of honeybees are all closely related to skeletal development; therefore, we hypothesize that selection on calcitonin receptor protein genes plays an important role in honeybee bone tissue development and evolution; however, further experiments of gene function are needed for validation.

#### 4.3.2 Genes related to reproduction

Reproduction-related traits are economically important traits in the Chinese honeybee. In alpine regions, colony reproduction only lasts for about 6–8 weeks, making it imperative for colonies to expand within a narrow window of time in the spring (Xu et al., 2018b). This is necessary for rapid collection during the short period of heavy nectar flow, storing a sufficiently large amount of food and developing large colonies to increase overwintering rates. In this study, we detected enrichment for various GO entries, such as “meiosis I cell cycle process” and “homologous recombination” and these genes (putatively under selection) may contribute to high reproductive rates (Figure 4; Supplementary Tables S6, S8). Among these GO terms, we identified *MAPK* (*APICC_00669*) and *MAST* (*APICC_03515*) as candidate genes related to honeybee reproductive traits and environmental adaptation. First, both *MAPK* and *MAST* are members of the serine/threonine protein kinase family, with important roles in regulating a series of physiological and biochemical responses, such as cell proliferation, cell differentiation, and apoptosis (Liang and wan, 2019). The MAPK pathway is an important part of growth and development, with roles in embryonic development and organ formation. A transcriptomics-based analysis revealed that more than 50% of the genes in the MAPK signaling pathway are differentially expressed during honeybee ovary activation and egg laying (Chen, 2018). Highly phosphorylated *MAST2* and *MPK3* in worker bee embryos regulate the latency of cell proliferation (Xiao et al., 2013). The JNK pathway mediated by MAPK is involved in ovarian differentiation and sex formation in zebrafish (Xiao et al., 2013). Selection on *MAST* and *MAPK* in the Yungui Plateau-type Chinese honeybee may favor a high reproductive capacity in the species. Thus, long-term adaptation may have resulted in a high egg-laying capacity, large spleen area, and fast reproduction.

#### 4.3.3 Genes related to temperature adaptation

In insects, thermoregulation is a great challenge in adaptation to high-altitude climates (Hodkinson, 2005). To cope with the cold season, bees have evolved a unique mechanism of social cooperation; numerous bees gather in groups, consume stored honey, and vibrate their flight muscles at high frequencies to generate heat and maintain the hive temperature (Coelho, 1991). For individual bees, cold tolerance involves regulating their metabolism (e.g., by activating the “glucose-glycerol-amino acid” system) (Xu et al., 2018a; Su et al., 2022). Glucose, lipids, and amino acids are important energy sources, and the metabolism of energy sources not only provides heat but also generates energy for long-distance flight. In this study, many genes under selection were significantly enriched for “Pentose and glucuronate interconversions,” “Fructose and mannose metabolism,” “Glycerolipid metabolism,” “Biosynthesis of amino acids,” and “positive regulation of intracellular signal transduction” (*p* < 0.05) (Supplementary Table S8), which further suggests that honeybees resist environmental stress by positively regulating the metabolism of energy substances and by working as a team. This may be a key mechanism by which honeybees adapt to cold environments.

Heat shock factor (HSF) regulates the response to various environmental stresses and initiates the expression of heat shock protein genes (Yu et al., 2012; Murshid et al., 2018). In this study, *HSF* (*APICC_09465*) was identified as a selected gene; we hypothesized that this gene was associated with the resistance of Chinese honeybees to the cold climate of the Yungui Plateau. Heat shock proteins (HSPs) are synthesized in large quantities within a short period of time after an individual organism is subjected to stress (Kanagasabai et al., 2011; Zininga et al., 2018). They are often used as markers of cellular stress (Nicewicz et al., 2021). HSPs play an important role in the adaptation of honeybees to environmental changes (King and MacRae, 2015); their expression is induced under high temperatures (Liu et al., 2014; Al-Ghzawi et al., 2022), cold (Xu et al., 2017) and electromagnetic field stress (Molina-Montenegro et al., 2023), conferring stress tolerance. In addition to stress tolerance, fecundity and longevity have been linked to the expression of HSPs (Hoffmann et al., 2003; Vermeulen and Loeschcke, 2007). In addition, the previously discussed MAPK family plays important roles in insect resistance to changes in temperature. For example, MAPK plays an important role in the response to low-temperature domestication and low-temperature-induced stunting in insects, such as *Bemisia tabaci* (Li et al., 2012), *Bombyx mori* (Fujiwara and Denlinger, 2007), and the western honeybee (Xu, 2021). The p38MAPK signaling pathway mediated by *MAPKK6* has a role in resistance to oxidative stress in Chinese honeybee (Wang, 2020). We detected the signature of selection in *MAPKK* (*APICC_02754*) in this study and hypothesized that this gene is heavily utilized in the long-term resistance of western honeybee populations to harsh ecological environments. Further work is needed to explore gene functions and the contribution of observed genetic variation to honeybee adaptation to different environments.

Overall, based on a functional enrichment analysis of genes under selection, we hypothesized that the Yunnan-Guizhou Plateau-type Chinese honeybee population adapted to the food resources and climatic environment of the Plateau region mainly via the regulation of growth and development, reproduction, and cold resistance. We identified *CTR*, *MAPK*, *MAST*, *HSF*, *MKKK*, and other key candidate genes associated with the adaptation of Chinese honeybees to the Yunnan-Guizhou Plateau environment. These genes provide a basis for future studies of the mechanisms underlying the environmental adaptation of Chinese honeybees to different habitats and responses to major climatic events.

## 5 Conclusion

We investigated the genetic structure and diversity of Chinese honeybee (*Apis cerana*) populations in Guizhou, China using whole-genome resequencing, revealing a certain degree of genetic differentiation between populations in eastern and western Guizhou. Local ecological differences are important factors leading to the observed genetic differentiation in this species. Using genome-wide scans for selection, candidate genes for adaptation to the environment of the Yunnan-Guizhou Plateau were identified. This study further provides a reference for the conservation and genetic improvement of native Chinese honey bee genetic resources in Guizhou Province, and helps us to understand the mechanism of Chinese honey bee adaptation to different habitats.

## Data Availability

The data presented in the study are deposited in the SRA repository, accession number PRJNA1054499.
