# Supplementary material for: Whole-genome resequencing reveals genetic diversity and adaptive evolution in Chinese honeybee (Apis cerana cerana) in Guizhou, China
Source: Front Genet. 2024 May 17;15:1352455. doi: 10.3389/fgene.2024.1352455 (PMC11140131; doi:10.3389/fgene.2024.1352455)
Supplement: Supplementary file 1 [file Table2.DOCX]

Supplementary Table S1 Bee sample collection information table

| Sample code | sampling region | Sample size | Sampling location | Longitude | Latitude | Elevation |
| --- | --- | --- | --- | --- | --- | --- |
| NP | Niupeng | 10 | Fahong Village, Niupeng Town | 103° 46' 19" E | 27° 07' 15" N | 1816.4 |
| ZS | Zhongshui | 10 | Huahongyuan Village, Zhongshui Township | 103° 50' 53" E | 27° 13' 19" N | 2071.0 |
| CS1 | Chishui 1 | 10 | Malu Village, Lianghekou Township | 105° 44' 0" E | 28° 21' 28" N | 866.5 |
| CS2 | Chishui 2 | 10 | Shibao Township Hongxin Village | 106° 10' 45" E | 28° 30' 39" N | 549.8 |
| WC1 | Wuchuan 1 | 10 | Huangyang Village, Daping Street | 108° 1' 5" E | 28° 39' 49" N | 929.0 |
| WC2 | Wuchuan 2 | 10 | Zhennan Township concentric village | 107° 55' 34" E | 28° 43' 43" N | 745.6 |
| SM | Shimen | 10 | Quanfa Village, Shimen Township | 104° 48' 0" E | 27° 0' 0" N | 2247.9 |
| SL | Shilong | 10 | Caohai Township Shilong Village | 104° 11' 4" E | 26° 47' 42" N | 1964.8 |
| ZA1 | Zheng'an 1 | 10 | Furongjiang Township Jianshan Village | 107° 26' 59" E | 28° 29' 27" N | 655.0 |
| ZA2 | Zheng'an 2 | 10 | Miao Ding Village, Hexi Township | 107° 20' 18" E | 28° 24' 25" N | 1138.4 |
| HS | Heishi | 10 | Heishi Township Horizontal Village | 104° 4' 51" E | 26° 47' 21" N | 2482.3 |
| XS | Xueshan | 6 | Baimao Village, Xueshan Township | 104° 4' 51" E | 27° 11' 53" N | 2370.7 |

Supplementary Table S2 Data quality control and reference genome alignment results

| Sampling location | Clean Base/Gb | Q30 /% | GC content /% | Comparison ratio /% | Sequencing depth /× | Degree of coverage /% |
| --- | --- | --- | --- | --- | --- | --- |
| ZS | 22.60 | 92.39 | 35.02 | 96.50 | 9.03 | 95.97 |
| SL | 22.83 | 92.27 | 34.35 | 96.63 | 8.93 | 97.44 |
| HS | 23.78 | 91.83 | 35.02 | 96.74 | 9.26 | 96.78 |
| XS | 14.06 | 91.76 | 33.43 | 96.56 | 8.83 | 98.86 |
| CS2 | 23.78 | 92.15 | 34.47 | 96.77 | 9.18 | 97.23 |
| CS1 | 22.39 | 92.22 | 35.47 | 95.43 | 8.96 | 95.41 |
| SM | 22.59 | 92.28 | 34.83 | 96.68 | 8.90 | 96.67 |
| ZA2 | 23.96 | 92.43 | 34.95 | 96.63 | 9.36 | 96.69 |
| ZA1 | 23.02 | 92.43 | 33.35 | 96.41 | 8.79 | 98.80 |
| WC1 | 23.60 | 91.75 | 33.55 | 93.10 | 8.64 | 99.15 |
| NP | 23.79 | 91.12 | 33.04 | 96.36 | 9.11 | 99.54 |
| WC2 | 23.71 | 91.77 | 32.54 | 93.47 | 8.59 | 99.60 |

Note: The sampling place codes, NP, Niupeng; ZS, Zhongshui; HS, Heishi; XS, Xueshan; SM, Shimen; SL, Shilong; CS1, Chishui 1; CS2, Chishui 2; ZA1, Zheng'an 1; ZA2, Zheng'an 2; WC1, Wuchuan 1; WC2, Wuchuan 2.

Supplementary Table S3 SNP statistical results

| Category | Number of SNPs |
| --- | --- |
| Upstream | 25126 |
| Exonic Stop gain | 144 |
| Exonic Stop loss | 21 |
| Exonic Synonymous | 39786 |
| Exonic Non-synonymous | 11030 |
| Intronic | 370743 |
| Splicing | 342 |
| Downstream | 19088 |
| upstream/downstream | 1313 |
| Intergenic | 932837 |
| Total | 1400430 |

Supplementary Table 4 Error rate of cross validation of genetic structure analysis of 116 samples

| K | Cross-validation error |
| --- | --- |
| 2 | 0.50059 |
| 3 | 0.53300 |
| 4 | 0.55890 |
| 5 | 0.59566 |
| 6 | 0.62530 |
| 7 | 0.66894 |
| 8 | 0.68830 |
| 9 | 0.72877 |


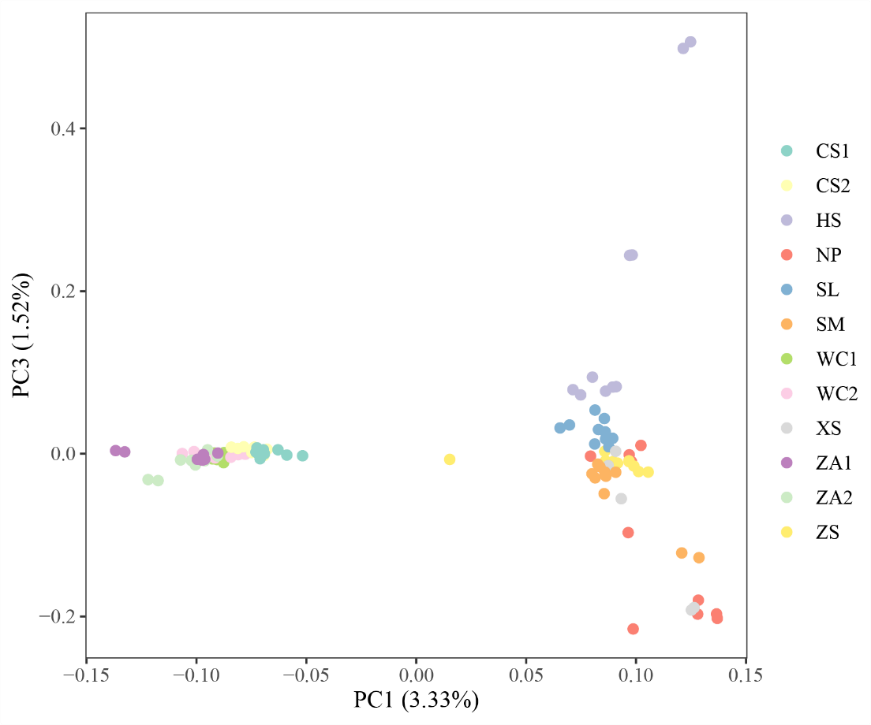


Supplementary Figure S1.

Scatter plot principal components 1 versus 3 (PC1 versus PC3). NP, Niupeng; ZS, Zhongshui; HS, Heishi; XS, Xueshan; SM, Shimen; SL, Shilong; CS1, Chishui 1; CS2, Chishui 2; ZA1, Zheng'an 1; ZA2, Zheng'an 2; WC1, Wuchuan 1; WC2, Wuchuan 2.


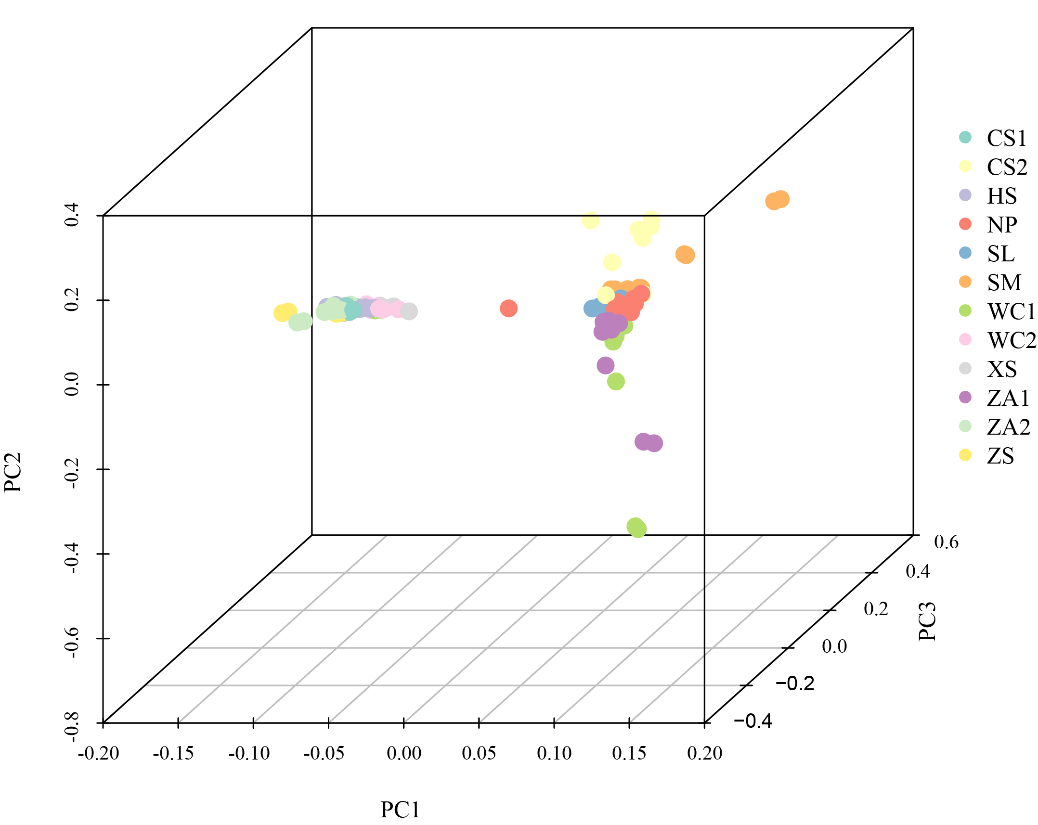


Supplementary Figure S2

Scatter plot principal components 1，2 versus 3 (PC1,PC2versus PC3). NP, Niupeng; ZS, Zhongshui; HS, Heishi; XS, Xueshan; SM, Shimen; SL, Shilong; CS1, Chishui 1; CS2, Chishui 2; ZA1, Zheng'an 1; ZA2, Zheng'an 2; WC1, Wuchuan 1; WC2, Wuchuan 2.


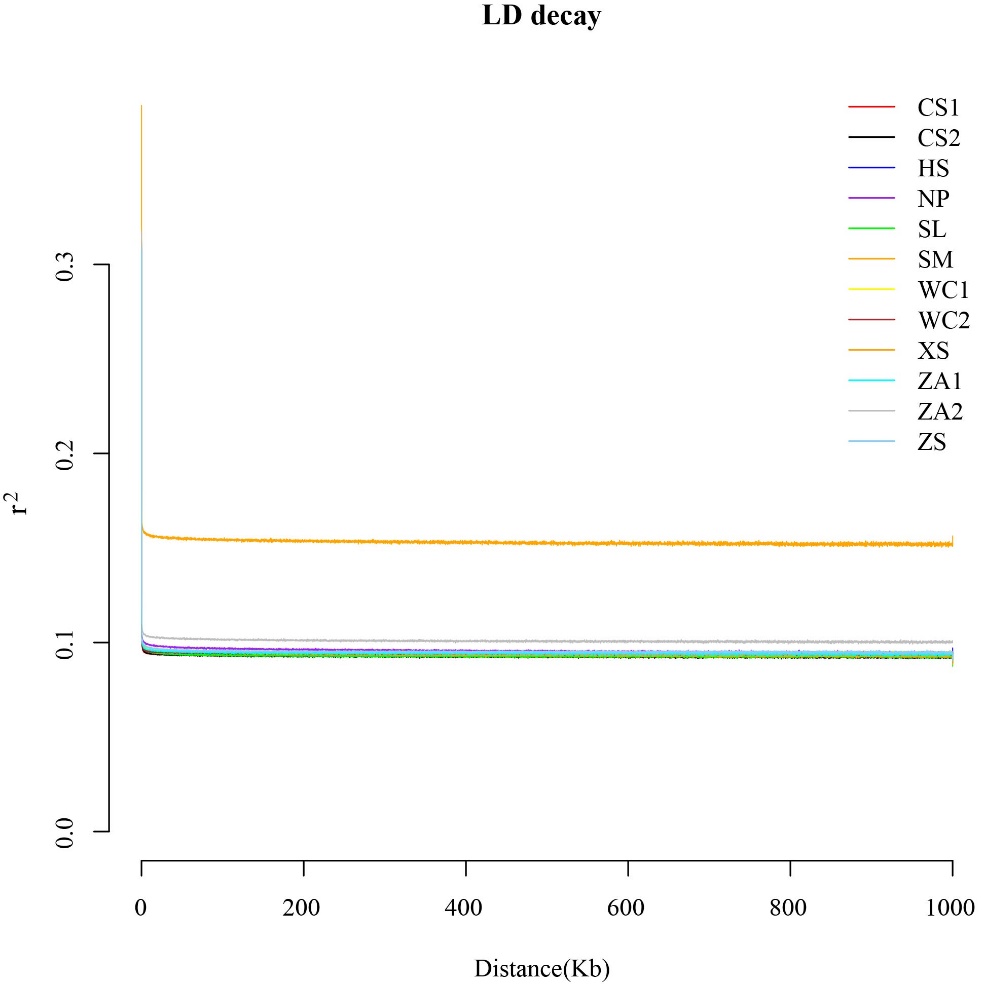


Supplementary Figure S3

Decay of linkage disequilibrium (LD) in Chinese honey bee populations in 12 geographic regions of Guizhou, China, with one line for each species. The vertical coordinate of r^2^ is the square of the allele frequency correlation. The horizontal coordinate is the attenuation distance.


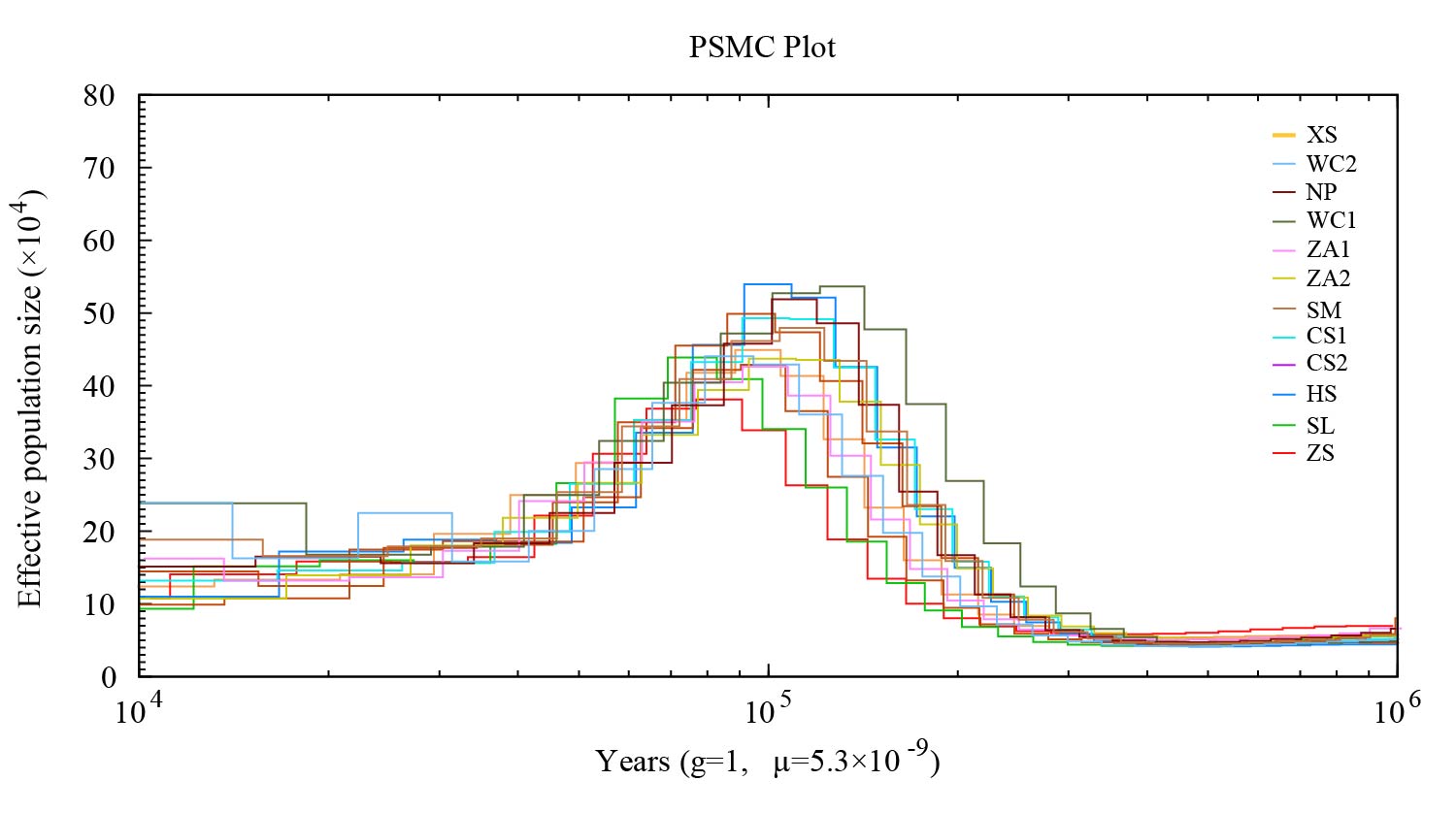


Supplementary Figure S4

Estimation of historical effective population sizes. g is the time in years it takes for the species to reproduce for one generation. μ is the mutation rate of the species.


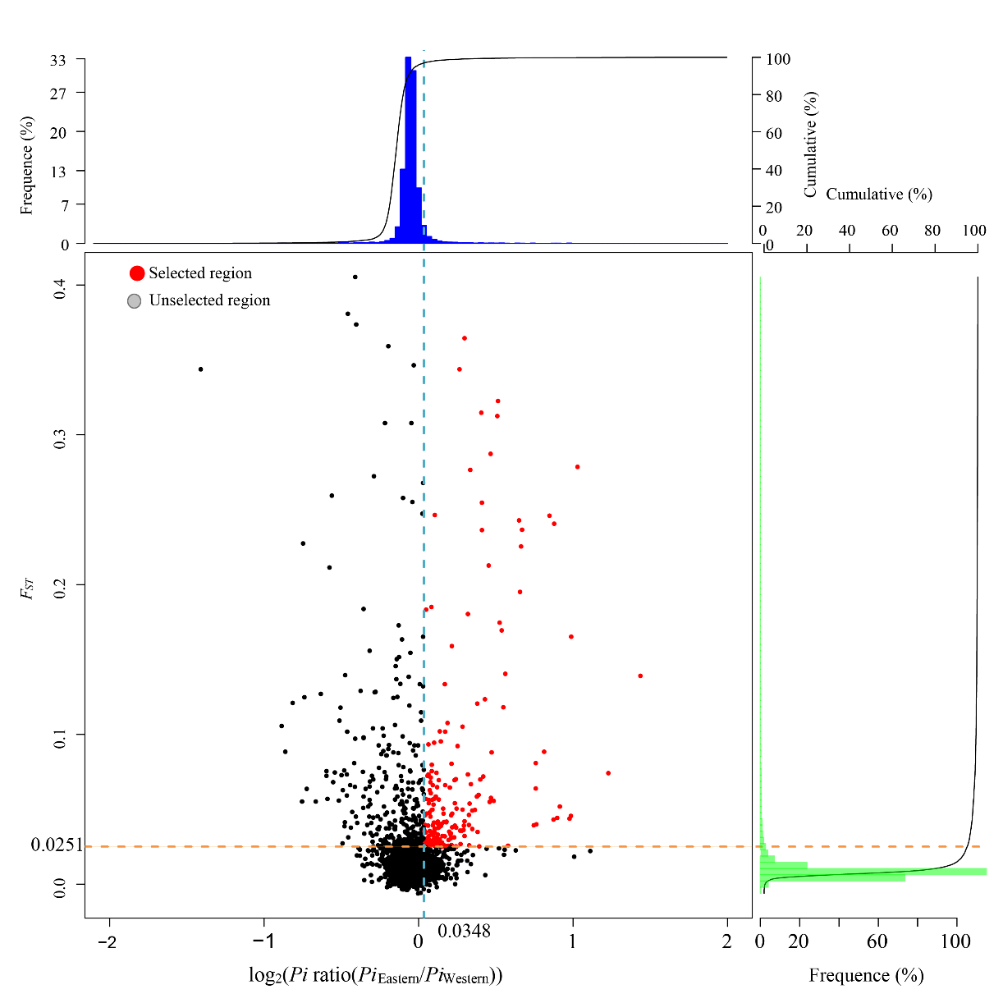


Supplementary Figure S5

Selective scanning analysis of western Guizhou populations against reference eastern populations**.** Selected intersections in the red region are in the top 5% based on *F_ST_* and log_2_ (*Pi* _Eastern_ /*Pi* _Western_).


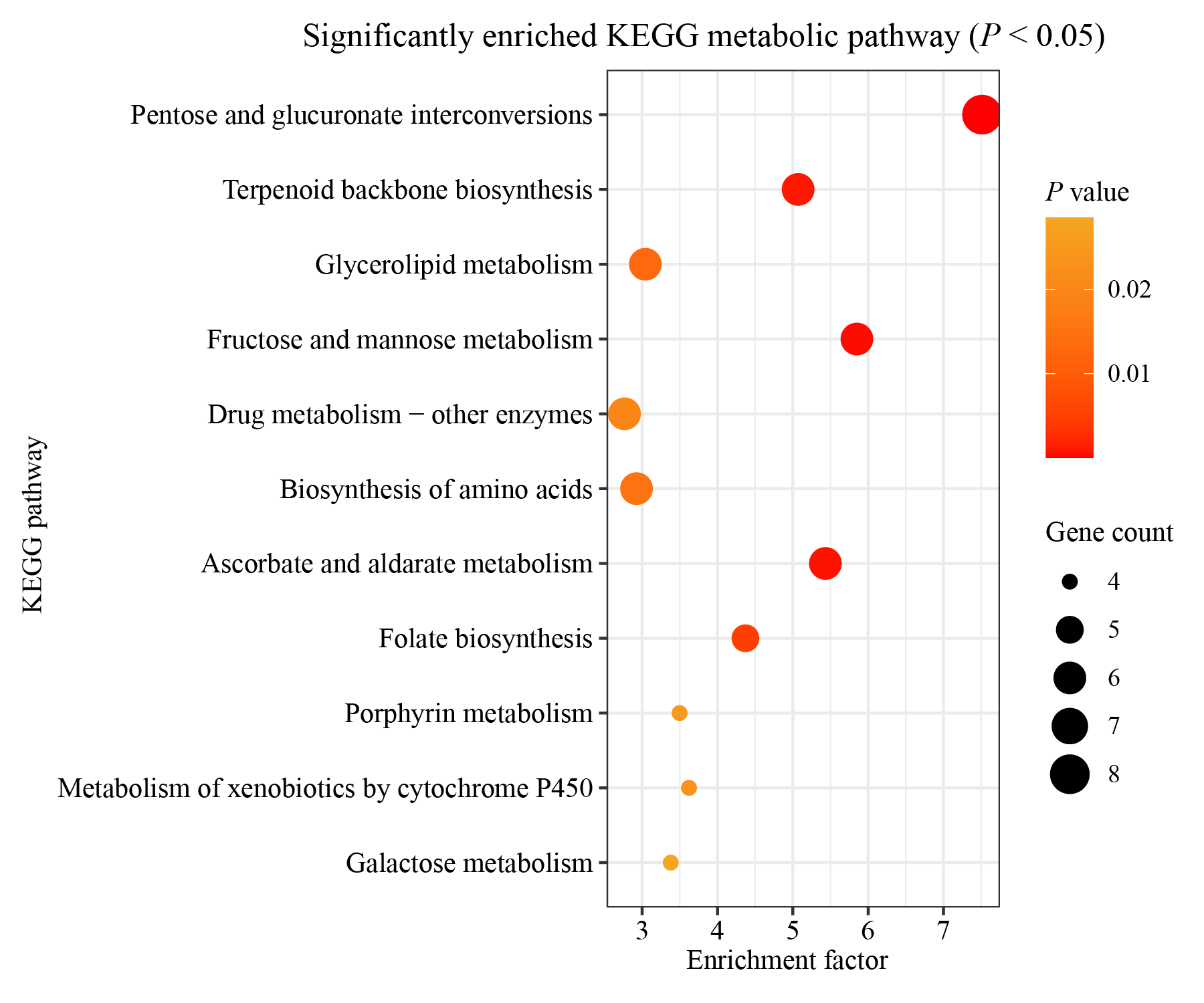


Supplementary Figure S6

Significant enrichment of KEGG metabolic pathway (*P* < 0.05). The size of the circle in the graph reflects the number of genes in that enriched pathway; the larger the circle, the greater the number of genes enriched. The color of the circle indicates the significance of the enrichment, the redder the color the higher the significance. The enrichment factor is the ratio of the number of selected genes annotated in a pathway to the number of all genes annotated in that pathway.
